# Bleeding and Pain Post-tonsillectomy: An Adult Cohort Study

**DOI:** 10.7759/cureus.80905

**Published:** 2025-03-20

**Authors:** Elliot Heward, John Rocke, George McNally, Sian Dobbs, Safdar Sarwar, Sara Timms, Sadie Khwaja, Nirmal Kumar

**Affiliations:** 1 Otolaryngology Department, Wrightington, Wigan and Leigh NHS Foundation Trust, Wigan, GBR; 2 Otolaryngology Department, Northern Care Alliance NHS Foundation Trust, Manchester, GBR; 3 Otolaryngology Department, Manchester University NHS Foundation Trust, Manchester, GBR; 4 Otolaryngology Department, Bolton NHS Foundation Trust, Bolton, GBR

**Keywords:** peri-operative analgesia, post-operative bleeding, post-operative care, post-operative pain, tonsillectomy

## Abstract

Background

Tonsillectomy is one of the most common surgical procedures performed. This study aimed to understand the rate and severity of post-operative bleeding and pain in adult patients undergoing tonsillectomy in the United Kingdom.

Methods

A total of 10 secondary and tertiary otolaryngology units recruited adults prior to tonsillectomy and provided them with a diary to complete and return. This diary recorded the frequency and severity of post-operative bleeding and the intensity and pattern of post-operative pain.

Results

In total, 75 patients were recruited with 18 complete patient diaries analysed (median age 24.8, range: 17-50 years); of these 12 (67%) reported post-operative bleeding. Pain was most severe on Days 4-6 (median score 8/10) and then declined over the 21-day study period. There were 21 post-operative interactions with healthcare services recorded by these patients: 15 emergency department and six general practice visits.

Conclusion

This study demonstrates the pattern of post-procedural pain and bleeding in adult tonsillectomy and the high rate of interaction with healthcare providers. Improved understanding will allow more accurate consent conversations and patient counselling.

## Introduction

Tonsillectomy is one of the most common procedures performed by ear, nose and throat (ENT) specialists [1]. Despite this, little is known regarding the post-operative morbidity that these patients experience, due to limited prospective data. In the latest Getting it Right First Time (GIRFT) National Speciality report, Hospital Episode Statistics (HES) demonstrated that the readmission rate following tonsillectomy in adults was 18.4% (range: 9.2-31.25%) [2]. These admissions are largely a result of post-operative pain or bleeding and have a negative impact on patient experience and bed capacity. Finding ways of reducing readmissions has therefore come into sharp focus [3].

Without a true understanding of the pattern of post-operative pain and bleeding following tonsillectomy, it is challenging to counsel patients accurately during the consent process regarding their expected experience. This deficiency in knowledge is likely to precipitate further interactions with healthcare services [4]. This prospective study aims to investigate the post-operative course of patients undergoing tonsillectomy; to understand the incidence and severity of post-operative pain and bleeding.

## Materials and methods

Reporting guideline

Strengthening the Reporting of Observational Studies in Epidemiology (STROBE) guidelines were used for this manuscript.

Study design

**Figure 1:**
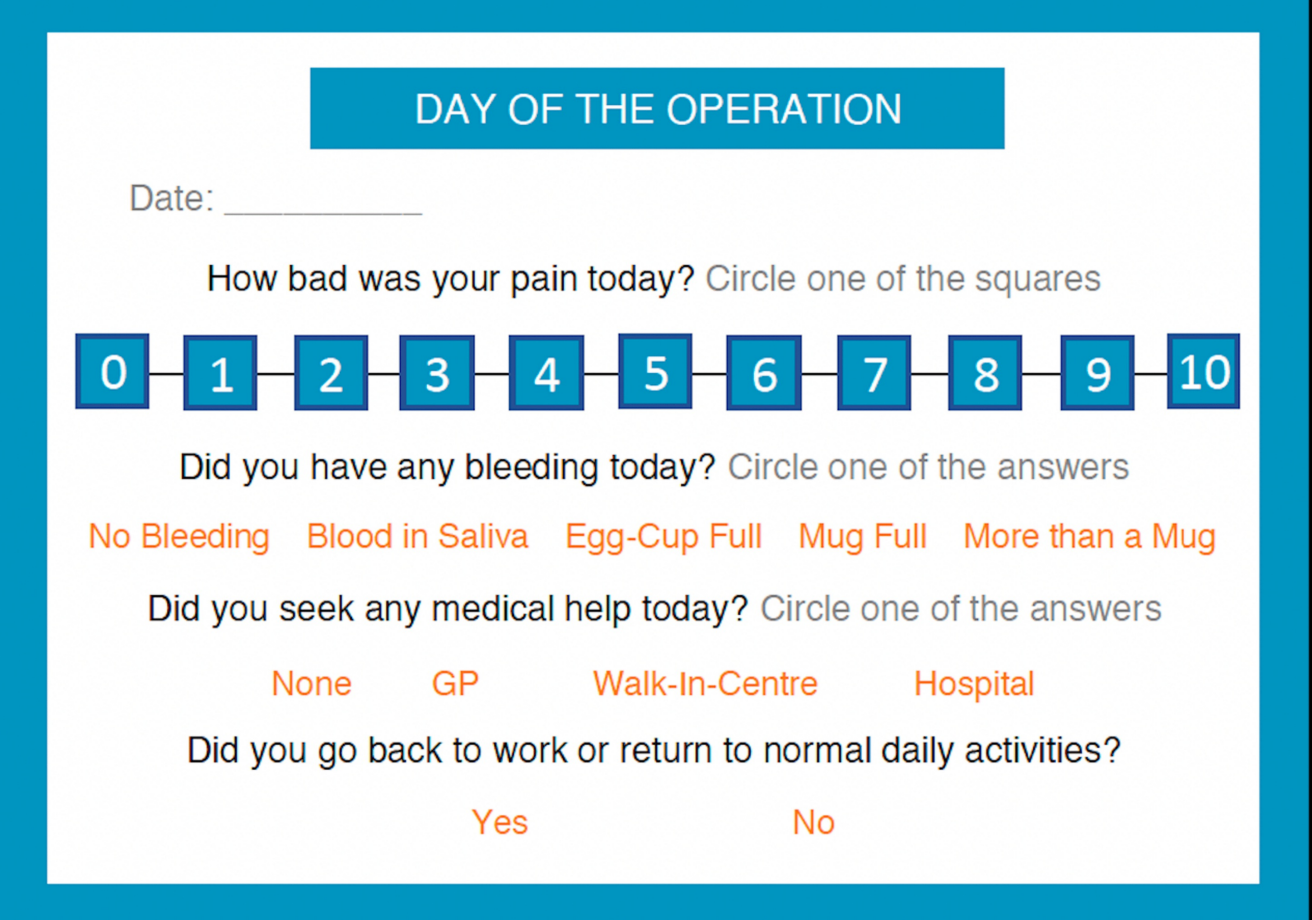
Post-tonsillectomy diary Note: This image was created by the authors for this study.

A diary was designed in collaboration with Patient and Public Involvement (PPI) members and the North West of England Otolaryngology Collaborative Research Group (Figure 1). There is no standardised measurement instrument which is validated for recording post-tonsillectomy bleeding quantity. Therefore, service users and otolaryngology doctors created a pragmatic and acceptable measurement scale to categorise bleeding quantity: no bleeding, blood in saliva, egg cup full, a mug full, and more than a mug. Approximate measurements which correspond with the bleeding categories are: egg cup (40ml), mug full (300ml), and more than a mug (>300ml). Pain severity was recorded using the Numerical Pain Rating Scale (NPRS) which is a recommended measurement tool by the British Pain Society [5]. The scale uses 0 = no pain and 10 = extreme pain/worst possible pain. The NPRS has been shown to have excellent test-retest reliability [6].

Patients provided consent and enrolled in this study on the day of their operation. Data was collected from the day of the operation (Day 0) until postoperative Day 21. Patients posted their completed diaries to the study team in a pre-paid envelope. The local study team collected patient demographics and surgical factors on an electronic case report form in Microsoft Excel (eCRF). This study received full ethical approval by the West of Scotland Research Ethics Service on the 22nd of January 2020 and the Health Research Authority on the 27th of January 2020 (20/WS/0013). All patients consented before recruitment into the study.

Setting

A total of 10 hospital sites were involved in this study: Royal Albert Edward Infirmary, Blackpool Royal Infirmary, Royal Preston Hospital, Fairfield General Hospital, Wythenshawe Hospital, Salford Royal Infirmary, Tameside General Hospital, Royal Bolton Hospital, Royal Blackburn Hospital and York Teaching Hospital. Patients were recruited from 1st March 2020 to 30th June 2022.

Participants

All male and female patients aged ≥17 years old who underwent bilateral tonsillectomy, using both extracapsular and intracapsular techniques, with or without adenoidectomy were included. Patients undergoing additional procedures or tonsillectomy for histology were excluded.

Bias

The electronic case report forms (eCRFs) ensured standardised data was collected in relation to the operative technique. There are no validated measures for reporting blood loss, therefore one was created by PPI members and the study management group. A numerical pain scale was used which is validated for use in adults [6]. To encourage participants to return their diary the study team called the patient two weeks following their operation as a reminder.

Statistical methods

Data was aggregated on Microsoft Excel (Microsoft Corp., Redmond, WA). Descriptive analysis was performed due to the low sample size.

Sample size calculation

The primary outcome used was post-operative bleeding. The incidence of adult post-operative bleeding of any nature has been shown to be between 7.8-11.9% [7-8]. This study used a post-operative bleeding incidence of 10% to calculate the sample size. A Clopper Pearson formula was used to calculate a 95% confidence interval with a 15% confidence interval width. A sample size of patients returning completed diaries was estimated at 74 patients. The dropout-inflated sample size was calculated with 70% dropout at 247 patients. This was based on the experience of the study sponsor, PPI group and collaborative research group. The recruitment aim was not met during the recruitment period due to recruitment constraints during the COVID-19 pandemic.

Patient and public involvement

The PPI Group at Wrightington Hospital helped design the study and create the patient diary. They were also involved in the interpretation of the study results.

## Results

Participants and descriptive data

The study recruited 75 patients (mean age 25.6 years, range: 17-50 years), of which 59 were female. A total of 18 (24.0%) completed diaries were returned for analysis (mean age 27.3, range: 17-50 years, females n=16). Recurrent tonsillitis was the most frequent indication (n=16/18). One patient had obstructive sleep apnoea and one patient had recurrent tonsilloliths. In terms of technique, the majority of patients underwent cold steel tonsillectomy (n=15/18), followed by bipolar tonsillectomy (n=2/18) and extracapsular coblation tonsillectomy (n=1/18). The lower pole was controlled with ties in all cases where cold steel was performed. No patients had an adenoidectomy. Codeine was prescribed to seven patients on discharge and no patients were discharged with morphine. No patients received infiltration of local anaesthetic agents. The tonsillectomy operations were performed by a foundation doctor (n=1), core surgical level doctors (n=5), registrar level doctors (n=9), and consultants (n=3).

Post-operative bleeding

**Figure 2:**
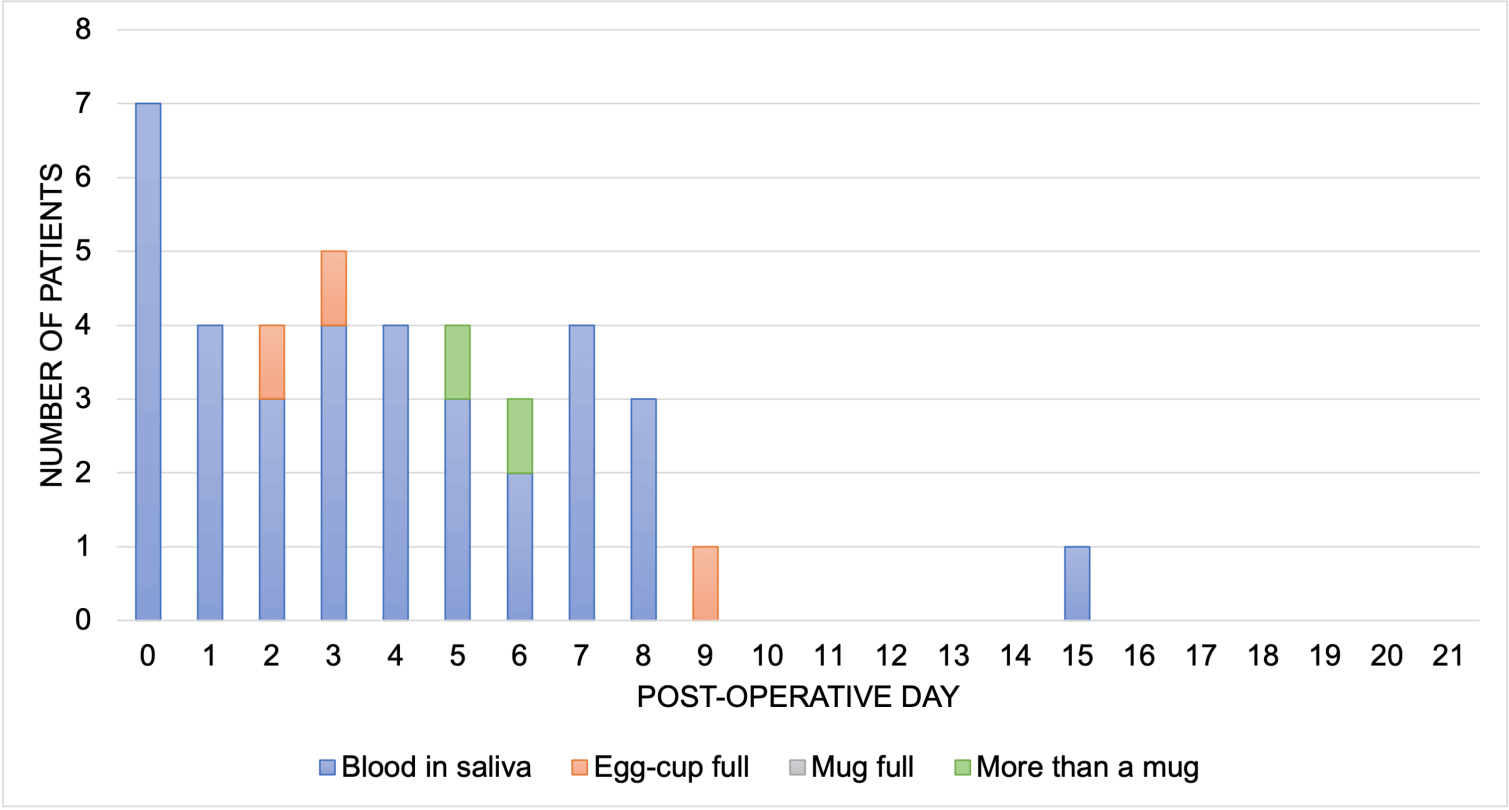
Bleeding following tonsillectomy by post-operative day

Post-operative bleeding was reported by 12 (67%) patients for a median of 2.5 days (range: 1-6 days) (Figure 2). Nine patients classified their worst bleeding episode as “blood in the saliva”, two patients as “egg-cup full” and one as “more than a mug full”. Bleeding was most frequent on the day of surgery. In all but one case, bleeding occurred within the first nine post-operative days.

Post-operative pain

**Figure 3:**
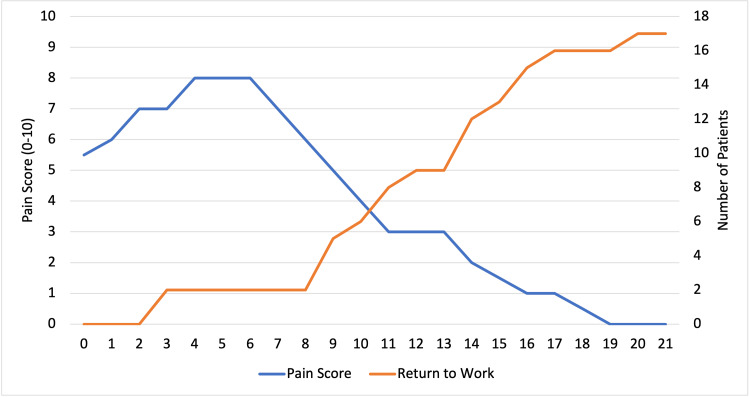
Median post-operative pain against return to work following tonsillectomy by post-operative day

Pain following tonsillectomy was 5.5/10 on the day of the operation and most severe on post-operative Days 4-6 (median 8/10) (Figure 3). The pain gradually declined over the three-week post-operative period. The median pain score for those who were prescribed codeine on Day 5 was 8/10 compared to 9/10 for those without codeine.

Return to baseline activities, work or education

All but one patient returned to baseline activities, work or education by post-operative Day 21 (Figure 3). The patient who returned to work on post-operative Day 3 had a pain score of 5/10. The majority of patients were in employment (n=12/18), followed by full-time education (n=3/18), not employed (n=2/18) and self-employed (n=1/18).

Interaction with healthcare services

**Figure 4:**
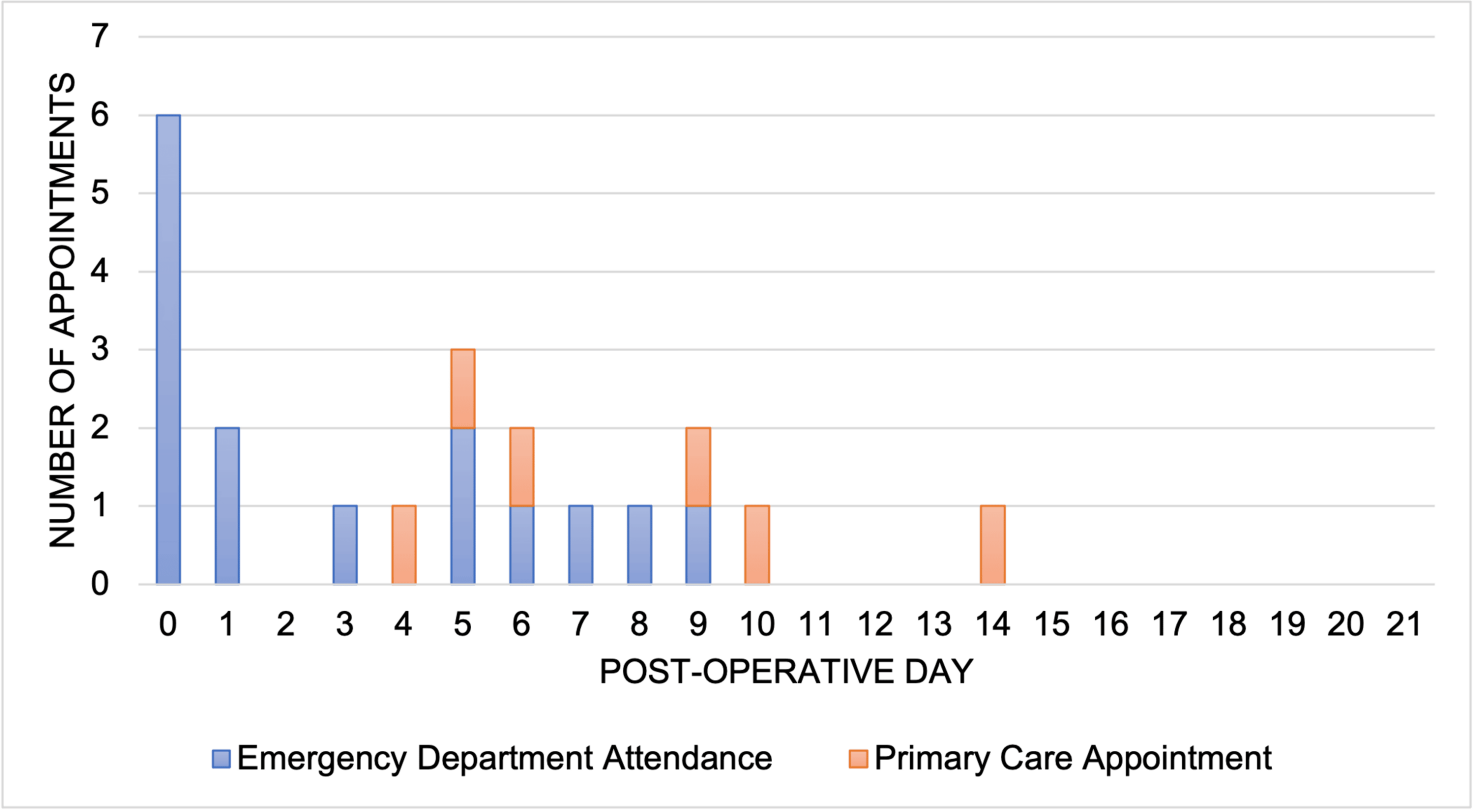
Interaction with healthcare services following tonsillectomy by post-operative day

A total of 12 patients (67%) attended health care services in the three weeks following their tonsillectomy. In total, 15 emergency department and six primary care appointments were required (Figure 4). All but one attendance occurred within the first 10 post-operative days.

## Discussion

This is the first prospective study to investigate the daily post-operative experience of adults undergoing tonsillectomy. This study demonstrates a high incidence of post-operative bleeding when compared to the national tonsillectomy audit of 2005, in which haemorrhage rates were quoted as 5.8% based on HES data [9]. This is a metric that has since been employed as an audit standard, for comparison of new techniques in research and during the consent process for patients undergoing tonsillectomy [10]. In our cohort study, the rate of bleeding was 67%, with patients bleeding for approximately 2.5 days. Low-volume bleeding (blood in saliva) comprises the majority of cases and may not necessitate medical review or admission in appropriately counselled patients. This rate is similar to a telephone survey that identified a bleeding rate of 61% in their survey of 99 patients [11]. The rate of post-operative bleeding in the paediatric population has been shown to be less frequent (44%) compared to adults [12].

The pattern of post-operative pain demonstrated a peak at Days 4-6 followed by a slow decline. The average pain score had not returned to zero when our study period ended on Day 21. Only 67% of patients felt their pain had returned to baseline 14 days after surgery. Return to work appears to coincide with reducing pain scores. Patients begin to return to work from post-operative Day 8. 

Our cohort of 18 patients required 21 further interactions with healthcare services in the three weeks after surgery. This is much higher than previous re-admission figures from the 2005 national audit and demonstrates the extra, previously unknown, burden that this procedure exerts on the healthcare system [9]. The high rate of low-volume post-operative bleeding may contribute to this finding. Appropriate counselling may help mitigate the requirement for post-operative healthcare interactions. 

Current tonsillectomy-related research is focused on comparing post-operative complications and recovery for different surgical techniques. Intracapsular coblation tonsillectomy appears to be a favourable technique in children [12-14]. There is currently insufficient data to determine the preferable surgical technique in adults [15]. The current study failed to recruit sufficient patients to enable comparison of techniques. Howitz et al. have outlined a randomised controlled trial in adults to address this research question [16].

A limitation of this study was the anticipated and observed high dropout rate due to un-returned diaries. Factors which should be considered in future studies to improve diary return rate include: reduced diary duration, return incentives and alternative diary forms (e.g. electronic). This study failed to recruit sufficient participants to reach the sample size calculation which was due to the impact of the COVID-19 pandemic on routine operations in the UK. The pandemic may have also impacted the diary return rates. Of the small number of diaries returned, participants who experienced post-operative problems are more likely to return their diaries in turn skewing the results. 

It is challenging for patients to accurately quantify bleeding volume as it is rarely collected in a receiver. The method of classification used is subjective which increases variability. Therefore, we made no attempt to determine specific measured blood loss volumes. Future research should look to determine inter-patient variability in grading blood loss volumes using the classification system proposed in this study. 

## Conclusions

These results provide an insight into the post-operative recovery following tonsillectomy in adults. Improved understanding will allow more accurate consent conversations and patient counselling. Better patient awareness of the expected post-operative course will help prevent unnecessary re-attendance to healthcare services and improved patient experience. Future research should look to compare post-operative bleeding and pain rates against different surgical techniques in an adult population.
